# Genome-wide identification and expression analysis of the trihelix transcription factor family in sesame (*Sesamum indicum* L.) under abiotic stress

**DOI:** 10.1007/s11033-023-08640-w

**Published:** 2023-08-16

**Authors:** Yunyan Zhao, Junchao Liang, Zhiqi Wang, Tingxian Yan, Xiaowen Yan, Wenliang Wei, Meiwang Le, Jian Sun

**Affiliations:** 1 College of Agriculture, Yangtze University, Jingzhou, 434025 China; 2 Jiangxi Province Key Laboratory of Oilcrops Biology / Nanchang Branch of National Center of Oilcrops Improvement, Crops Research Institute of Jiangxi Academy of Agricultural Sciences, Nanchang, 330200 China

**Keywords:** Sesame, Trihelix transcription factor, Genome-wide analysis, Gene expression, Abiotic stress

## Abstract

**Background:**

The plant trihelix gene family is among the earliest discovered transcription factor families, and it is vital in modulating light, plant growth, and stress responses.

**Methods:**

The identification and characterization of trihelix family members in the sesame genome were analyzed by bioinformatics methods, and the expression patterns of sesame trihelix genes were assessed by quantitative real-time PCR.

**Results:**

There were 34 trihelix genes discovered in the genome of sesame, which were irregularly distributed among 10 linkage groups. Also, the genome contained 5 duplicate gene pairs. The 34 trihelix genes were divided into six sub-families through a phylogenetic study. A tissue-specific expression revealed that *SiTH* genes exhibited spatial expression patterns distinct from other trihelix genes in the same subfamily. The cis-element showed that the *SiTHs* gene promoter contained various elements associated with responses to hormones and multiple abiotic stresses. Additionally, the expression patterns of 8 *SiTH* genes in leaves under abiotic stresses demonstrated that all selected genes were significantly upregulated or downregulated at least once in the stress period. Furthermore, the *SiTH4* gene was significantly induced in response to drought and salt stress, showing that *SiTH* genes may be engaged in the stress response mechanisms of sesame.

**Conclusion:**

These findings establish a foundation for further investigation of the trihelix gene-mediated response to abiotic stress in sesame.

**Supplementary Information:**

The online version contains supplementary material available at 10.1007/s11033-023-08640-w.

## Introduction

Transcription factors (TFs) binding to a cis-regulatory element in promoter or enhancer DNA activate or repress target gene expression, affecting plant growth and development [1, 2]. The trihelix gene family belongs to the first known TFs reported in plants [3] and was named after the presence of a specific three-helix structure (helix-loop-helix-loop-helix) in the DNA structural domain; in addition, this family is known as a GT factor because its structural domain specifically binds to GT elements necessary for the light response [4, 5]. All plant trihelix subfamilies contain one or two conserved three-helix structural domains, although their C-terminal structural domains vary [6]. The conserved domains of the trihelix family are similar to the three α-helices (helix-helix-turn-helix) of the MYB gene family, but the amino acid sequence between the two α-helices is longer; thus, their three-dimensional conformations are different, allowing them to recognize different DNA elements and bind specifically to GT elements on the DNA sequence [7]. In accordance with the characteristics of its conserved structural domains, Ruth categorized the trihelix family into five subfamilies (GT-1, GT-2, SH4, GTγ, and SIP1) [6]. Recently, Yu discovered a new subfamily, GTδ, in tomato [8].

The trihelix family exists widely in terrestrial plants [9]. Preliminary studies have concentrated on the regulation of the light response [4, 10]. The GT-1 factor, the first trihelix member found in *Pisum sativum*, binds to the GT element in the light-induced *rbcS-3 A* gene promoter [11]. Previous studies demonstrated that trihelix genes are associated with numerous biological processes, such as the regulation of late embryogenesis, and the formation of the perianth, stomata, and seeds [6, 12, 13]. The identification and cloning of trihelix genes from different subfamilies revealed their essential role in coping with stressful environments [14]. Li found that *ASR3 (At2g33550*) in *Arabidopsis thaliana* exerted a negative effect on modulating immune-related responses [15]. Three rice trihelix genes (*OsGTγ-1*, *OsGTγ-2* and *OsGTγ-3* ) and two soybean trihelix genes (*GmGT-2 A* and *GmGT-2B*) are involved in cold, drought, and salt stress responses [16, 17]. *AtGT2L* expression in Arabidopsis was induced during cold and salt stress [18]. Furthermore, there was evidence that trihelix genes are engaged in the abiotic stress response of maize [19].

Sesame is an ancient and traditional oilseed crop that provides a major supply of plant oil. Sesame seeds are rich in fat and protein [20]. However, the occurrence of environmental pressures in recent years, including salt, drought, and other adverse conditions, severely diminished the yield and quality of sesame [21–23]. With the complete sequencing of the sesame genome, many TFs involved in biotic and abiotic stresses in sesame were identified, such as NAC [24], WRKY [25], BZIP [26], HD-ZIP [27], and MATE [28]. Presently, there is not a comprehensive genomic investigation of the sesame trihelix family yet. It is necessary to identify and analyze the trihelix transcription factor family in sesame due to its potential role in stress responses.

## Materials and methods

### Genome-wide identification of trihelix family in sesame

The sesame genome sequence and annotation information were downloaded from the Ensembl Plants database. (http://plants.ensembl.org/index.html). The trihelix protein sequence of Arabidopsis was downloaded from TAIR (https://www.arabidopsis.org/). BLASTp alignment of Arabidopsis trihelix protein sequences to sesame protein sequences, screened for high homology, with an E-value cut-off of 1 e^− 5^. The Hidden Markov Model (HMM) file (*PF13837*) was downloaded from the PFAM database (http://pfam.xfam.org/), and the sesame genome was searched by Simple HMM Search, a built-in plug-in of TBtools [29]. The sesame trihelix protein was screened with an E-value 1 e^− 5^ as the threshold. BLASTp and HMM searches for candidate proteins were conducted, their protein sequences were downloaded, and whether they contained conserved domains peculiar to the trihelix family in SMART (http://smart.embl-heidelberg.de/) and NCBI-CDD (https://www.ncbi.nlm.nih.gov/cdd) was confirmed. We eliminated proteins without typical domains. The molecular weight, isoelectric point, and amino acid size of sesame trihelix protein were predicted and determined on the ExPASy website (https://www.expasy.org). WoLF PSORT (https://wolfpsort.hgc.jp/) was utilized to predict the subcellular localization of trihelix proteins.

### Phylogenetic and homology analysis

ClustalW was used to accomplish multiple sequence alignment of trihelix family proteins. The best model was built with MEGAX(https://www.megasoftware.net), and the phylogenetic tree was created with the Neighbour-Joining (NJ) approach. The bootstrap parameter was set to 1000, while the other parameters were left at their default values. Use the online tool Evolview (https://evolgenius.info/evolview) to edit and enhance the phylogenetic tree image. The McScanX software in TBtools was used to examine collinearity among sesame trihelix genes, and an intraspecific collinearity diagram was drawn.

### Gene structure analysis and conserved motif identification

The GSDS (http://gsds.cbi.pku.edu.cn) online produced the *SiTH* gene structure. To compare the differences in *SiTHs*, the conserved protein motifs in sesame trihelix proteins were identified and analyzed by the online search program, MEME (http://meme.nbcr.net/meme/intro.html). The relevant parameters were set as follows: The width of the motif was 6 to 50 amino acids, and the number of motifs requested was 12. Subsequently, the resulting data was portrayed in TBtools.

### Analysis of cis-acting elements

TBtools software was used to extract the 2000 bp sequence upstream of the initiation codon of the trihelix gene. Plant Care (http://bioinformatics.psb.ugent.be/webtools/plantcare/html/), an online promoter analysis tool, was used for detecting cis-acting elements implicated in abiotic stress in the *SiTH* genes. TBtools software was used for visual display.

### Plant material growth and stress treatment

Sesame samples from different tissues and plump sesame seeds of “Zhongzhi 13”, were selected and sown in germination boxes. After being grown for 5 days in a greenhouse with a cycle of 16 h of light and 8 h of darkness, the seedlings could be transferred to a hydroponic tank and cultivated in 1/2 Hoagland nutrient solution. Roots, stems, and leaves were collected after 2 weeks of seedling growth. The tissues of flowers, capsules, and seeds were sampled during flowering. After freezing in liquid nitrogen, the samples were preserved in the refrigerator at − 80 °C for subsequent analysis. Stress treatment and sampling: The same two-week-old sesame seedlings were chosen for drought, salt, low temperature, and abscisic acid (ABA) protocols for preculture conditions. Stress treatment: Low temperature treatment transported sesame seedlings from the greenhouse to a lit incubator at 4 °C, whereas other treatments transported seedlings to 1/2 Hoagland nutrient solution with 150 mmol/L NaCl (salt stress), 15% PEG 6000 (drought stress), and 100 µM ABA. Sampling: take fresh sesame leaves at 0 h, 1 h, 2 h, 4 h, 8 h, 12 h, and 24 h; DEPC water washed leaves; dried by filter papers; placed in a 2.0 mL centrifuge tube; frozen in liquid nitrogen in a -80 °C refrigerator. Each treatment and time point had three biological duplicates taken.

### Total RNA extraction and quantitative PCR analysis

Total RNA was obtained from different tissues of sesame grown under control cultivation and leaf samples grown under abiotic stress (drought, salt, low temperature, and ABA). Total RNA used the Plant RNApure Kit (Zomanbio, Beijing) and detected by electrophoresis. The utilization of a reverse transcription kit allowed for the synthesis of cDNA (SR511, Beijing Jinsha Biotechnology Co., Ltd). The test instrument was LightCycler96 (Roche, USA), and Takara RR820A was used for qRT-PCR. Procedure extension referenced receptor instructions. Repeat genetically and biologically three times each. The *SiH3.3* gene was used as an internal reference gene. The 2^−ΔΔCT^ method served to calculate the relative gene expression levels under abiotic stress. The 2^−ΔCT^ method served to calculate the relative gene expression levels in different tissues [30]. A t-test was used to analyze the significance of relative expression data. The Primer3 Plus online website (Primer3Plus-Pick Primers) was used to design primers. Primers were synthesized by Bioengineering (Shanghai) Co., Ltd. Table S1 contains all of the primer information.

## Results

### Identification of trihelix gene family in sesame

34 trihelix genes were identified in the sesame genome by homologous BLAST and HMM domain searches. Table 1 summarized the characteristics of these trihelix genes, including gene ID, protein length, gene location, protein molecular weight (MW/KD), isoelectric point (PI), and sub-cellular localization. The predicted length of sesame trihelix proteins varied from 171 aa (SiTH19) to 826 aa (SiTH12), and their molecular weight ranged from 19.95 KD (SiTH19) to 91.80 KD (SiTH12). The isoelectric point of SiTH proteins ranged from 4.61 (SiTH10) to 9.83 (SiTH28). The subcellular localization prediction of 34 trihelix proteins showed that most SiTH proteins were located in the nucleus, 3 trihelix proteins (SiTH1, SiTH2, and SiTH12) were in the cytoplasm, SiTH4 was predicted to be in the peroxisome, and SiTH20 was predicted to be in the chloroplast.

**Table 1:** Basic information on trihelix family members in sesame

Name	Gene ID	Length/aa	Gene localization	MW/KD	PI	Sub-cellular localization
*SiTH1*	*SIN_1021687*	333	LG1:693312:696382	39.25	8.93	cytoplasm
*SiTH2*	*SIN_1021674*	281	LG1:799635:801679	31.73	4.73	cytoplasm
*SiTH3*	*SIN_1009584*	371	LG1:12034848:12035963	42.64	4.70	nucleus
*SiTH4*	*SIN_1010837*	343	LG1:13008779:13010375	38.89	5.86	peroxisome
*SiTH5*	*SIN_1008092*	330	LG1:16218620:16220342	36.44	8.93	nucleus
*SiTH6*	*SIN_1007508*	223	LG2:3859096:3859857	25.38	6.05	nucleus
*SiTH7*	*SIN_1013334*	437	LG2:4362049:4363362	50.55	5.98	nucleus
*SiTH8*	*SIN_1013333*	620	LG2:4385274:4387857	68.52	6.25	nucleus
*SiTH9*	*SIN_1018023*	401	LG2:18166468:18169978	45.14	5.99	nucleus
*SiTH10*	*SIN_1010262*	374	LG3:7886575:7887699	42.46	4.61	nucleus
*SiTH11*	*SIN_1017501*	351	LG3:9859259:9860314	39.29	9.04	nucleus
*SiTH12*	*SIN_1017304*	826	LG3:12040810:12056627	91.79	7.01	cytoplasm
*SiTH13*	*SIN_1008992*	437	LG3:24019030:24020343	50.37	6.01	nucleus
*SiTH14*	*SIN_1008991*	616	LG3:24031048:24033628	68.05	6.00	nucleus
*SiTH15*	*SIN_1005532*	414	LG5:681083:682327	46.85	9.26	nucleus
*SiTH16*	*SIN_1013518*	300	LG5:5647953:5648855	35.25	4.62	nucleus
*SiTH17*	*SIN_1016029*	577	LG5:8446816:8450842	65.91	8.13	nucleus
*SiTH18*	*SIN_1016078*	277	LG5:9284117:9288009	31.50	9.06	nucleus
*SiTH19*	*SIN_1002877*	171	LG5:11551650:11552288	19.95	7.85	nucleus
*SiTH20*	*SIN_1012581*	529	LG6:609684:611512	61.02	6.18	chloroplast
*SiTH21*	*SIN_1018587*	742	LG6:12159445:12164508	81.36	5.62	nucleus
*SiTH22*	*SIN_1020903*	459	LG6:16317944:16321559	51.73	6.61	nucleus
*SiTH23*	*SIN_1020555*	270	LG6:18727409:18729768	31.78	8.26	nucleus
*SiTH24*	*SIN_1027105*	336	LG8:7794900:7795910	38.09	5.71	nucleus
*SiTH25*	*SIN_1026536*	384	LG8:12266352:12270509	43.13	6.11	nucleus
*SiTH26*	*SIN_1022988*	379	LG8:17870321:17871460	43.83	5.34	nucleus
*SiTH27*	*SIN_1022989*	431	LG8:17873852:17875147	49.43	6.07	nucleus
*SiTH28*	*SIN_1017662*	207	LG10:7272014:7272637	22.90	9.83	nucleus
*SiTH29*	*SIN_1009862*	341	LG11:2244576:2245601	37.85	9.48	nucleus
*SiTH30*	*SIN_1012982*	529	LG11:11940705:11943295	59.77	6.70	nucleus
*SiTH31*	*SIN_1012955*	482	LG11:12120109:12121557	54.69	6.12	nucleus
*SiTH32*	*SIN_1011076*	280	LG11:14748067:14749515	33.12	7.69	nucleus
*SiTH33*	*SIN_1006772*	337	LG12:5893280:5894859	37.49	8.55	nucleus
*SiTH34*	*SIN_1025352*	524	LG15:7008853:7010825	58.95	5.50	nucleus

### Chromosome localization and gene replication analysis

**Fig. 1:**
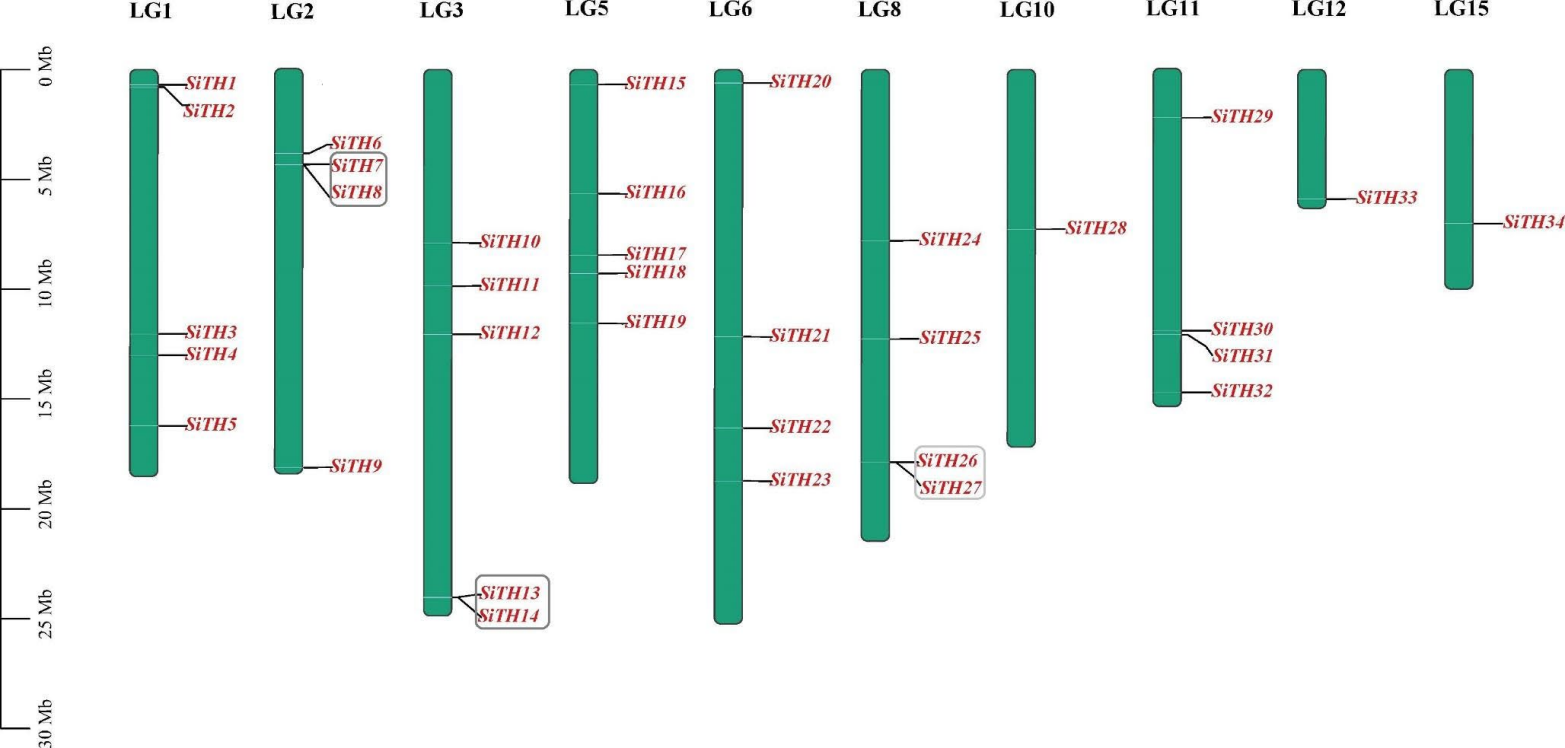
Chromosome locations of sesame *SiTH* family members. Green bars represent linkage groups (LGs). The LG number was shown above the bar. Trihelix genes are labelled at the right of the LGs. The scale bar denotes the LG lengths (Mb)

According to information on chromosome annotation, 34 trihelix genes of sesame were unequally distributed in 10 linkage groups (LG) (Fig. 1). Each linkage group contained 1 to 5 genes. Among these groups, the LG1, LG3, and LG5 linkage groups contained five trihelix genes, whereas the LG10, LG12, and LG15 linkage groups contained only one *SiTH* gene. No *SiTH* genes were detected in the LG4, LG7, LG9, LG13, LG14, and LG16 linkage groups.

**Fig. 2:**
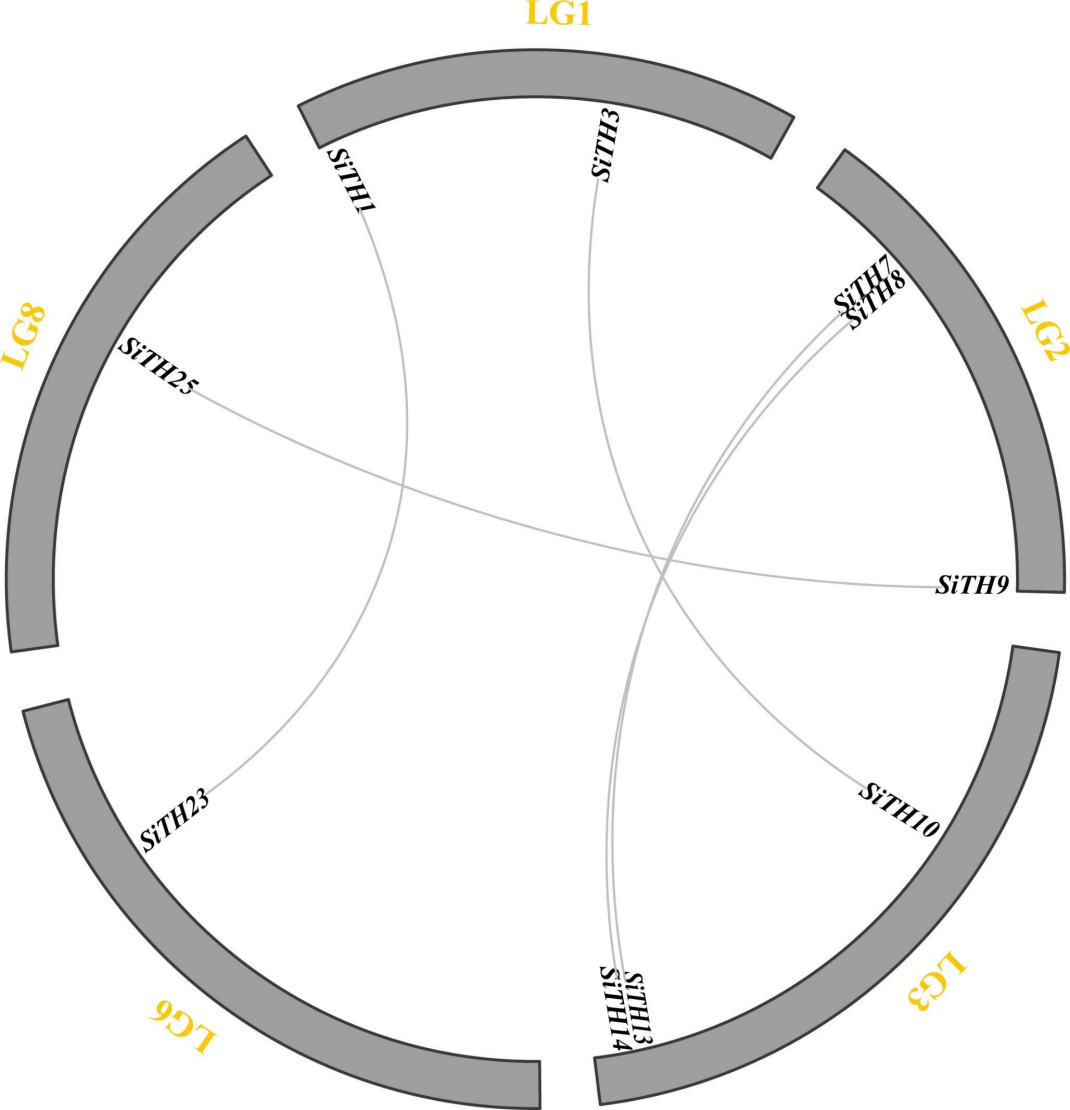
Genome distribution of segmentally duplicated trihelix gene pairs in sesame. Hair lines indicate pairs of duplicated trihelix genes

To reveal the evolutionary mechanism of the trihelix gene family in sesame, duplication events were analyzed. The results demonstrated that the trihelix gene family underwent tandem and segmentary duplication. Three pairs of trihelix genes in sesame were identified as tandem duplicates (Fig. 1). Five pairs of *SiTHs* had segmental repeat events, as shown in Fig. 2. This result indicated that repeated gene events played an important role in amplifying the sesame trihelix gene family.

### Phylogenetic analysis

**Fig. 3:**
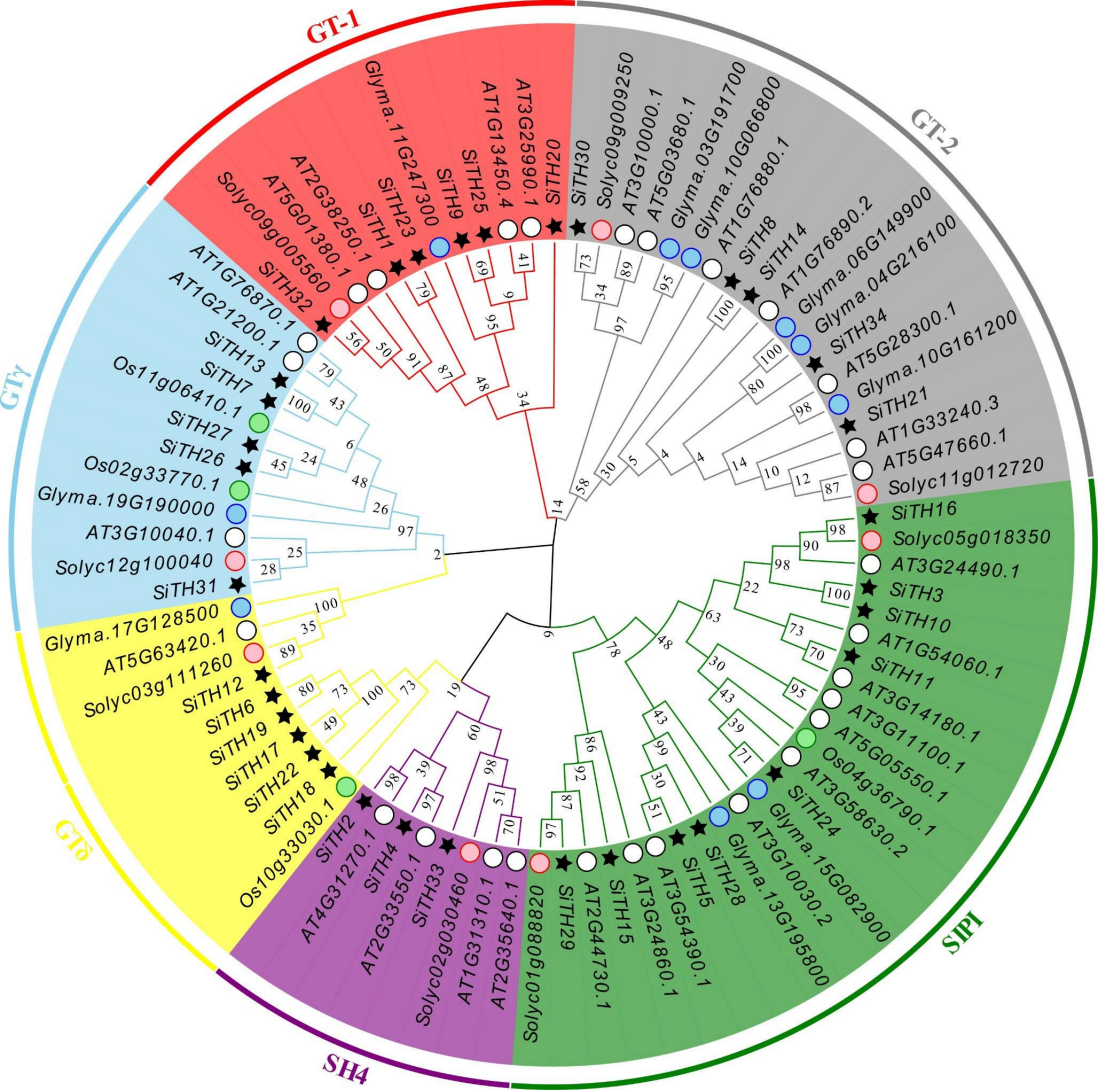
Phylogenetic analysis of 86 *trihelix* proteins from *Arabidopsis*, rice, tomato, soybean and *Sesamum indicum* L. The phylogenetic tree was built with MEGAX, then edited and beautified with Evolview, an online tool. The white circle, green circle, pink circle, sky blue circle and black star represent trihelix proteins in *Arabidopsis*, rice, tomato, soybean and *Sesamum indicum* L., respectively

To reveal the phylogenetic relationship of the sesame trihelix family, we generated a phylogenetic tree from the amino acid sequences of trihelix proteins from sesame and other crops (Arabidopsis, tomato, soybean, and rice) by MEGAX software. The analysis found that the *SiTH* genes were classified into six subfamilies (Fig. 3), including SIP1, GTγ, GTδ, GT-1, GT-2, and SH4. Similar findings were also found in Arabidopsis and tomato [8]. SIP1 was the largest subfamily of sesame, containing 9 *SiTH* genes. The smallest group was the SH4 subfamily, which contained 3 *SiTH* genes. Moreover, the numbers of *SiTHs* in the GTγ, GTδ, GT-1 and GT-2 subfamilies were 5, 5, 7 and 5, respectively. The phylogenetic tree showed that the genetic similarity between *SiTH7* and *SiTH11* were highly similar (approaching 100%) in the GTγ subfamily. In the GT-2 subfamily, *SiTH8* and *SiTH14* exhibited strong similarities. In the SIP1 subfamily, the similarity between *SiTH3* and *SiTH10* genes was also approximately 100%. These gene pairs showed a closer relationship compared to others, indicating that they may perform similar functions directly.

### Gene structure and conserved motif analysis

**Fig. 4:**
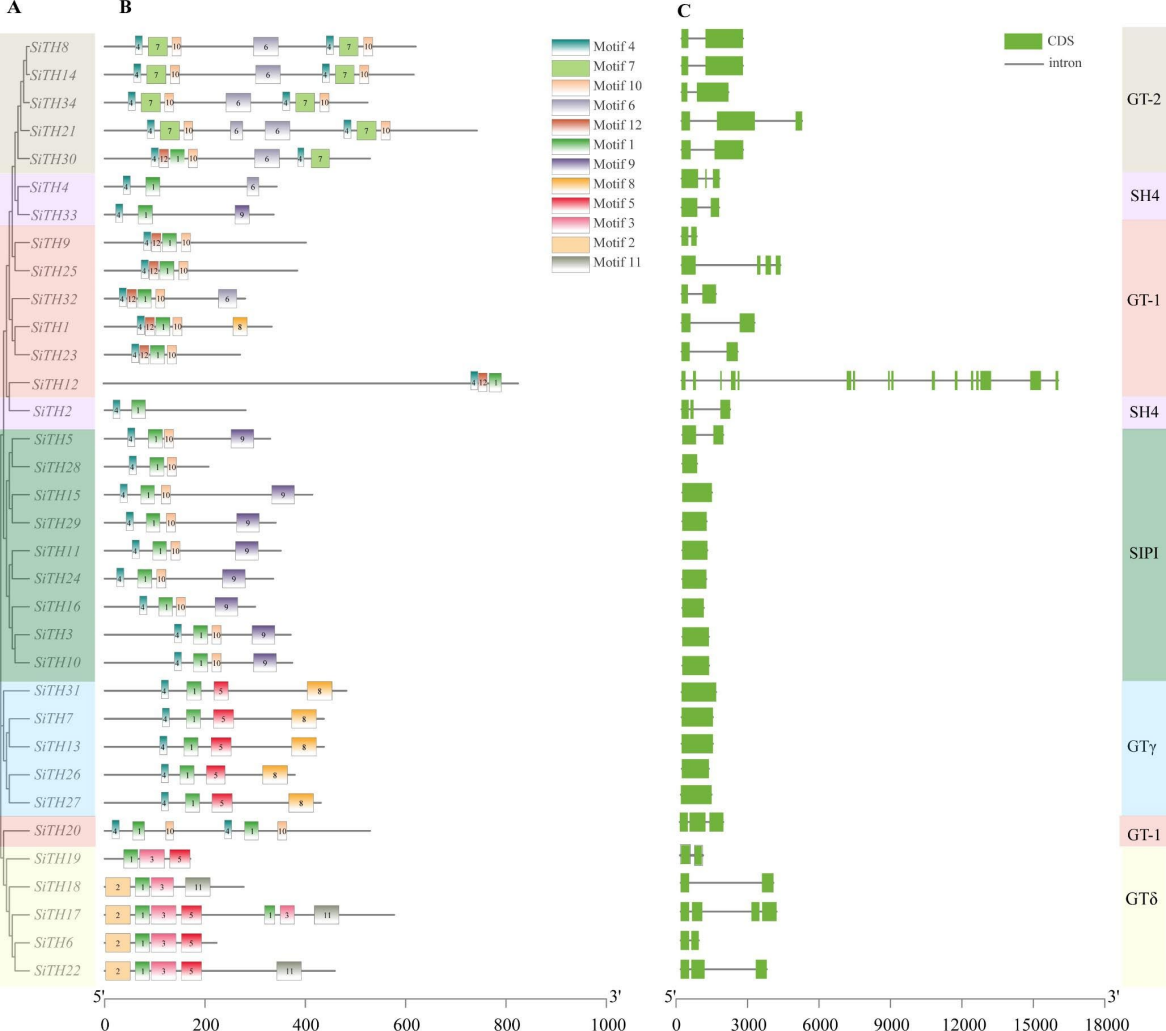
The conserved motifs and gene structure of sesame trihelix family. **A** Phylogenetic relationships of the SiTH proteins. **B** Putative motifs in the 34 SiTH proteins. Conserved motifs were identified using MEME. The length of each protein could be estimated using the scale at the bottom. **C** The structures of 34 *SiTH* genes. CDS and introns are indicated by black lines and green boxes, respectively

To identify the differences among trihelix family genes in sesame, we evaluated their gene structure and conserved motifs. The number and distribution of exons and introns of 34 sesame trihelix genes were shown in Fig. 4C. The number of exons of 34 *SiTH* genes was discontinuously distributed from 1 to 16. More than half of the CDS of *SiTH* genes were separated by introns. Most of the assembled trihelix members had a similar ratio of exons to introns (Fig. 4A), and the intron trihelix transcription factor family members were all from the GTγ and SIP1 subfamilies, which was similar to the identification results in *Fagopyrum tataricum* and rice [9, 31]. All members of the GTγ subfamily contained one exon. *SiTH5*, a member of the *SIP1* subfamily, contains 2 exons, and the other 8 members contained only one exon. Except for *SiTH21*, GT-2 subfamily members contained two exons. SH4 subfamily members contained 2 to 3 exons, and GTδ subfamily genes usually included 2 to 4 exons. *SiTH23* in the GT-1 subfamily had the largest number of exons, containing 16. *SiTH25* had 4 exons and other members contained 2 to 3 exons. This indicated that there are some differences in the structure of *SiTH* genes.

To further analyze the origin and evolutionary pattern of SiTHs, we identified 12 conserved motifs in sesame SiTH proteins using the MEME program. As shown in Fig. 4B, most SiTH proteins contained motif 1 and motif 4. SH4 subfamily members contain motifs 1, 4, and 6 (SiTH6 lacks motif 6). SiTH23 lacked motif 12, while other GT-1 subfamily members contained motifs 1, 4, 10, and 12, the SiTH1 protein contained motif 8, and SiTH32 contained motif 6. Motif 2 and motif 11 only exist in the GTδ subfamily, and most GTδ subfamily members contained motifs 1, 2, 3, and 5. The SiTH members of the GT-2 subfamily contained motifs 1, 2, 3, 5, and 9. Interestingly, all members of this subfamily had two motifs 4 and 7, which was consistent with the characteristics of the GT-2 subfamily containing two trihelix domains. The conserved motifs of all member proteins in the GTγ subfamily were motifs 1, 4, 5, and 8. This means that members of the same subfamily had similar conserved motif compositions, and there were differences between different subpopulations, which might represent different functions of the trihelix family in sesame.

### Analysis of cis-acting elements of SiTHs

**Fig. 5:**
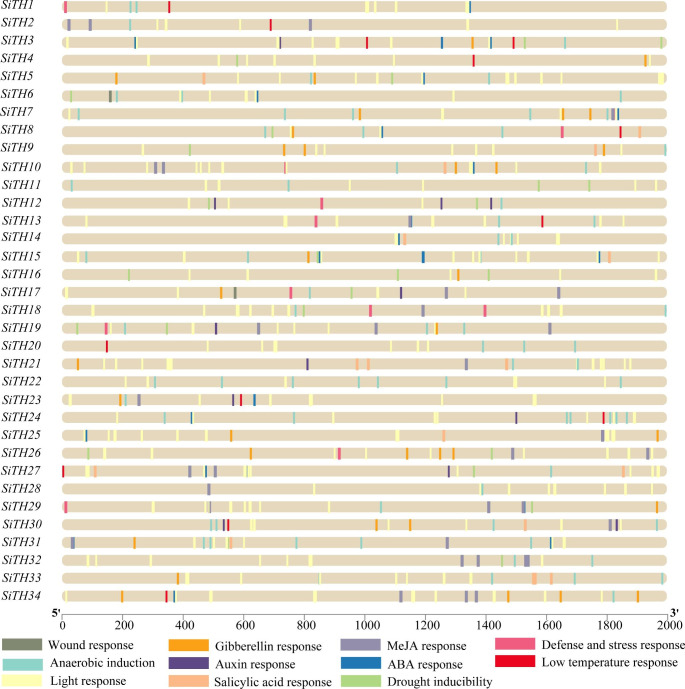
Predicted cis-elements in the promoter regions of the sesame trihelix genes. The scale bar at the bottom indicates the length of the promoter sequence

Cis-acting elements are involved in the regulation of gene expression[32, 33]. To explore the possible mechanism by which *SiTH* genes regulate expression, the 2000 bp sequence upstream of the start codon of trihelix gene in sesame was extracted, and the potential cis-acting elements in the promoter region were screened by Plant Care online tool. We predicted 11 types of cis-acting elements in sesame *SiTHs*, and element information is shown in Table S2 and Table S3. Figure 5 show that 79.4% of *SiTHs* contain more than 5 cis-elements. All *SiTHs* contained light responsive elements, which was consistent with the involvement of the trihelix gene family in the light response. Anaerobic inducible elements were more widely distributed than other abiotic stress-related elements, and 88.2% of *SiTHs* contained at least one anaerobic inducible element. Wound responsive elements, defense and stress responsive elements, low temperature responsive elements and drought inducible elements were detected in the promoter regions of 3, 11, 18 and 12 *SiTH* genes, respectively.

Additionally, hormone-responsive elements were detected, such as ABA responsive elements, gibberellin responsive elements, auxin responsive elements, MeJA responsive elements, and salicylic acid responsive elements. Among them, 24 genes contained ABA-related response elements, GA responsive elements were detected in 21 members, MeJA responsive elements were detected in 18 members, SA responsive elements were detected in 12 members, and auxin responsive elements were detected in 9 members. Moreover, some promoter elements were peculiar, such as wound response elements, which were only identified in *SiTH6*, *SiTH17* and *SiTH21*. The promoter region of the trihelix gene family of sesame had a variety of cis-acting elements related to hormonal and abiotic stress, indicating that the trihelix family gene might be involved in the growth and development of sesame. Moreover, they might participate in the regulation of various abiotic stress responses.

### Tissue-specific expression profiling of SiTHs

**Fig. 6:**
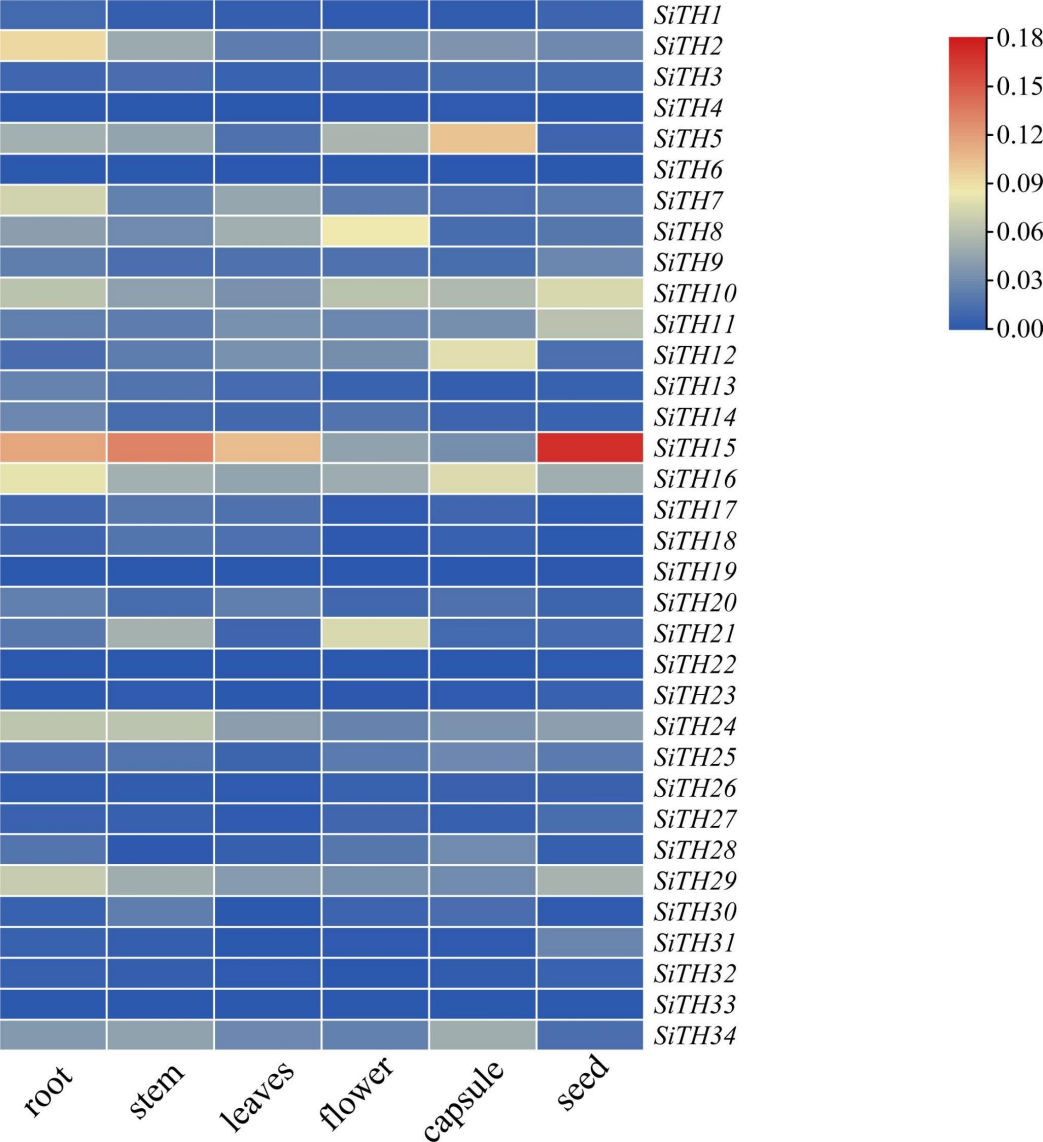
Heatmap of relative expression levels of the *SiTH* genes in different tissues. The relative expression data was obtained from qRT‒PCR calculated by using *SITH3.3* as the internal reference gene. The mapping data are shown in Table S4

To analyze the expression of *SiTHs* in various tissues and organs, we measured the expression profiles of *SiTH* genes under normal growth conditions in six tissues (root, stem, leaf, flower, seed, and capsule) of sesame by qRT-PCR. The heatmap showed that the expression levels of *SiTH* genes were lower than those of the internal reference genes, but the expression patterns were different in different tissues (Fig. 6). The relative expression levels of *SiTH15* in roots, stems, leaves and seeds were the highest. The expression of *SiTH8* was the highest in flowers. *SiTH5* was dominantly expressed in the capsule. The relative expression levels of 10 (*SiTH1, SiTH3, SiTH4, SiTH9, SiTH22, SiTH23, SiTH26, SiTH32* and *SiTH33*) genes in 6 different tissues were relatively low. The expression patterns of *SiTH* genes family members in sesame tissues were not consistent, which provided a reference for further study of the role of *SiTH* genes in sesame growth and development.

### **Analysis of** ***SiTH*** **expression patterns under abiotic stress**

**Fig. 7:**
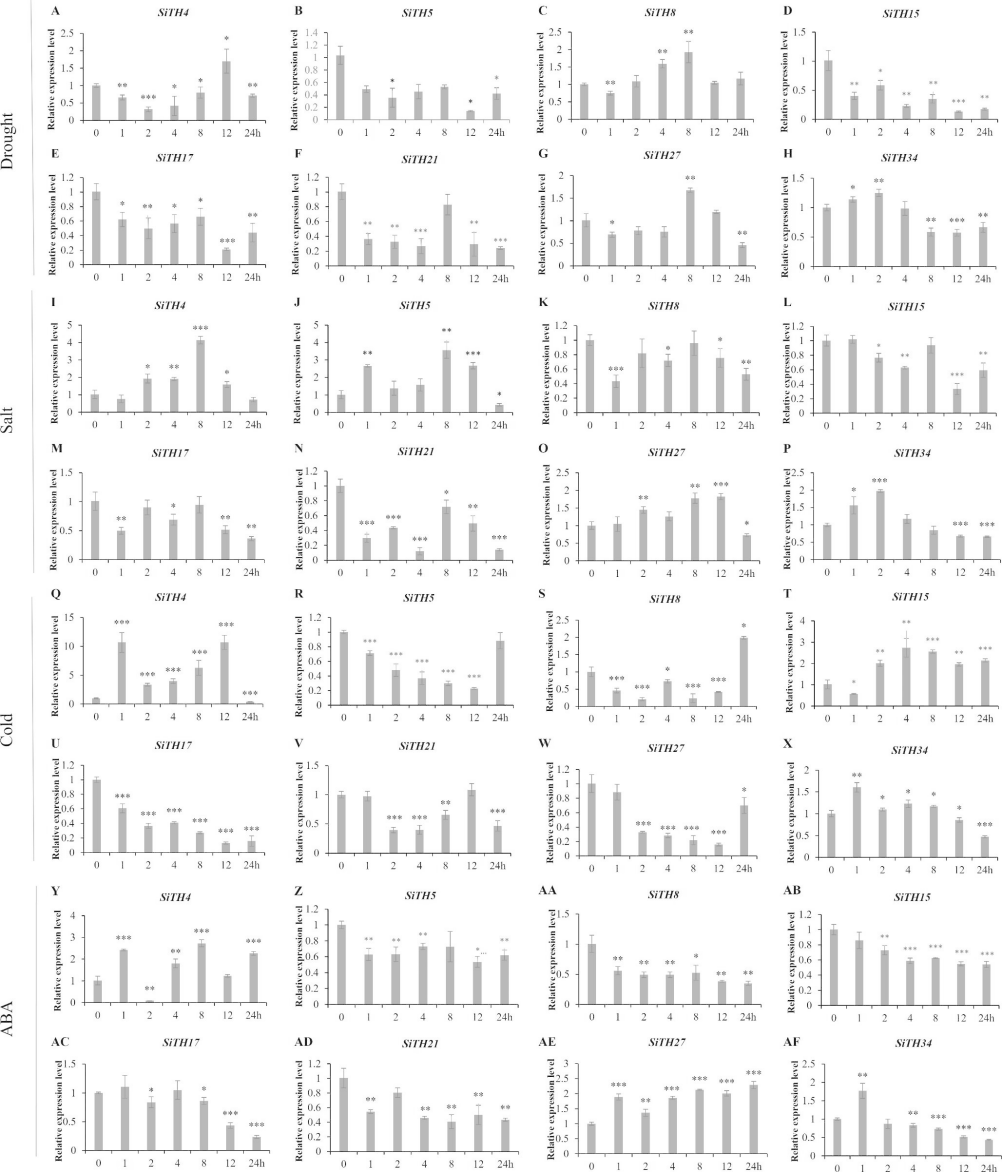
The relative expression levels of 8 selected trihelix genes under abiotic stress as revealed by qRT‒PCR. (**A–H**) The relative expression levels of selected genes under drought treatment; (**I–P**) The relative expression levels of selected genes under salt treatment; (**Q–X**) The relative expression levels of selected genes under cold treatment; (**Y–AF**) The relative expression levels of 8 *SiTH* genes under ABA treatment. Statistical significance was determined using t-tests (***p < 0.001, **p < 0.01, *p < 0.05, n = 3)

To preliminarily identify the expression pattern of *SiTHs* under abiotic stress, 8 genes were selected for qRT-PCR detection according to the results of evolutionary tree classification and cis-acting element analysis. The results of drought treatment (Fig. 7A-H) showed that *SiTH4* gene expression was upregulated at 12 h; *SiTH8* was upregulated at 2 h, 4 h, 8 h, and 12 h, and the upregulation was significant at 4 and 8 h. *SiTH34* was upregulated at 1 and 2 h, *SiTH27* expression was significantly upregulated at 8 h, and the expression of other genes was downregulated by drought treatment. The results of salt stress treatment (Fig. 7I-P) showed that 4 genes (*SiTH4*, *SiTH5*, *SiTH27* and *SiTH3*4) were upregulated at almost time points, *SiTH4* and *SiTH5* genes reached upregulation peaks (approximately 4 times, 3.5 times) at 8 h, and other genes were downregulated in response to salt stress. Under cold treatment (Fig. 7Q-X), 4 genes (*SiTH4*, *SiTH8*, *SiTH15*, and *SiTH34*) were upregulated at least two time points, and *SiTH4* and *SiTH15* genes peaked at 1 and 12 h (approximately 7 times, 2.5 times, respectively). In summary, we found that the expression patterns of several *SiTH* genes under different abiotic stresses exhibit certain similarities. *SiTH4* and *SiTH34* were upregulated under drought, salt and cold treatment, while *SiTH17* and *SiTH21* were both down-regulated. However, the expression patterns of the same gene were different when participating in different stresses. *SiTH8* was significantly upregulated under drought and cold stress, but it was downregulated at the next time point under salt stress, and significantly downregulated at 1 h of treatment (about 2 times). *SiTH5* tended to be downregulated under drought and low temperature stress, and upregulated under salt stress. These results illustrate that these *SiTH* genes may play a crucial role in the response to multiple abiotic stresses in sesame.

Abscisic acid (ABA) is an important plant hormone involved in various processes of growth, development, and interaction between plants and the environment. *SiTH* genes are rich in ABA cis-acting elements. The 8 selected *SiTH* genes except *SITH17* included at least one ABA response element. *SITH27* contained the most ABA cis-acting elements (containing 7). Under ABA treatment, the expression of *SiTH4*, *SiTH27*, and *SiTH34* was induced at least one time point (Fig. 7Y-AF). The expression of the *SiTH4* gene peaked at 8 h and then decreased, but it was still at the upregulation level. The expression of the *SiTH34* gene was upregulated 1 h after ABA treatment, but it was downregulated at subsequent practice time points. The expression of *SiTH27* gene continued to be significantly induced with ABA treatment and peaked at 24 h. Other *SiTH* genes were downregulated at each processing time point.

## Discussion

### Characterization of the sesame trihelix gene

The trihelix gene family plays an important role in the regulation of plant light response [34], leaf development [35], stomatal regulation [36], seed development [13, 37, 38], and biotic and abiotic stress responses [39, 40]. In recent years, the trihelix transcription factor family has already been identified in many plants, and its functions have also been investigated [19]. In this research, bioinformatics analysis of the sesame trihelix gene family was undertaken for the first time. 34 trihelix members were identified in sesame, which were irregularly distributed among 10 linkage groups. The length of these SiTH proteins ranged from 171 to 826 aa, the isoelectric point was 4.61 to 9.83, and the molecular weight was 19.95 to 91.80 KD. 29 SiTH proteins were hypothesized in the nucleus, and the remaining 5 genes were in the peroxisome, cytoplasm, and chloroplast. These genes may have very different functions from other trihelix genes.

Gene duplication is a key process in the evolution of new genes. The development of novel gene functions in response to changing environmental conditions is facilitated by these duplications [41, 42]. In most cases, gene families are amplified primarily through the following mechanisms: tandem duplication and segmental duplication [43]. Segmental replication is the main driving force for gene family amplification. In this study, only 3 pairs of tandem genes and 5 pairs of segmental duplication genes (29.41%) were found among sesame trihelix members, this indicated that more than half of them may not be related. These results explained the differences in the structure and conserved motifs of different subfamilies of SiTH proteins to some extent. Similar phenomena also occur in other crops, such as 71 soybean trihelix genes containing only 13 pairs of segment repeat genes, and only 6 pairs of repeat genes were contained among the 41 rice trihelix genes [31, 44].

Phylogenetic analysis allows revealing gene evolution. The 34 *SiTH* genes were divided into 6 subfamilies, among which SIP1 was the richest subfamily, containing 9 *SiTH* genes. The members of the sesame trihelix family containing 1–2 introns accounted for 38.24% of the total, which was similar to the results in soybean [16]. Trihelix genes belonging to one subfamily have similar structures in sesame, such as the length and distribution of conserved motifs are consistent, which may be associated with specific functions. The difference before different subfamilies is large, which indicates that the trihelix gene is not close in evolution. Trihelix genes have been lost or expanded during the evolution of sesame, which creates a potential for the formation of new genes.

### Expression analysis of sesame trihelix genes

Trihelix gene family is engaged in plant growth. In this study, we obtained the expression profiles of members of the sesame trihelix gene family in various tissues by qRT-PCR. Compared with the internal reference genes, the expression levels of SiTH genes were lower, and the expression patterns of different tissues were different. Among all members, *SiTH15* exhibited the highest expression in four tissues (roots, stems, leaves, and seeds), indicating that it may take an active impact in sesame growth. *SiTH5* had the highest expression in capsule and very low expression in seed. Different tissue expression patterns of *SiTHs* mean that members of the sesame trihelix family may have different functions.

With the frequent occurrence of extreme climate, plants are facing more severe survival conditions such as drought, salinization, and low temperature [45]. Many transcription factors are involved in regulating stress tolerance in plants. The trihelix members have been demonstrated to be crucial in the regulatory mechanisms of abiotic stress tolerance, which includes resistance to conditions like drought, salt, and low temperature. In Arabidopsis, *AST1* (*At3g24860*) mediated salt and osmotic stress tolerance [46]; GT-2 like 1 (*AtGTL1*, *At1g33240*) inhibited *SDD1* to modify stomatal density and drought tolerance; GT-4 (*AT3G25990*) adjusted *Cor15A* for salt tolerance [47]. The first Ca^2+^/CaM^−^ binding trihelix transcription factor, *AtGT2* (*At5G28300*), regulated low temperature and salinity stress [18]. ABA, a common stress hormone, may cause plant responses to various stressors [48]. Recent studies have revealed that the trihelix gene may adopt to drought and salinity stress via the ABA pathway. *BnSIP1-1* mediated ABA synthesis, signal transduction, salt response, and osmotic stress. Overexpression of the *BnSIP1-1* gene decreased rapeseed seedlings sensitivity to drought and ABA stress [49]. In this research, we detected that 73.68% of *SiTH* genes contained ABA response elements. The trihelix gene may be involved in response to abiotic stress through multiple cis-acting element regulatory pathways. Further analysis of the expression patterns of eight sesame trihelix genes in response to abiotic stress showed that the *SiTH4* and *SiTH34* genes were activated under drought, salt and cold treatment, whereas the *SiTH17* and *SiTH21* genes showed downregulated. Most selected *SiTH* genes expressed under ABA stress, but their expression varied substantially at different processing periods.

## Conclusions

In the sesame genome, we identified 34 members of the trihelix gene family and clustered them into 6 subfamilies. The same subfamily genes usually have similar structures and conserved functional domains. Stress tolerance candidate genes were found by examining *SiTH* gene expression profiles under drought, salt, low temperature, and ABA stress. This study provides a basis for elucidating the function and molecular mechanism of trihelix genes in plant development and stress tolerance.

### Electronic supplementary material

Below is the link to the electronic supplementary material.

**Table S1:** Primer sequence for qRT‒PCR;

**Table S2**: Cis-acting elements identified in *SiTH* genes

**Table S3**: The predicted cis-elements in the promoter regions of the sesame trihelix genes

**Table S4**: Relative Expression profiles of the sesame trihelix genes in different tissues

## Data Availability

The datasets generated or analyzed during this study are available from the corresponding author on reasonable request.
